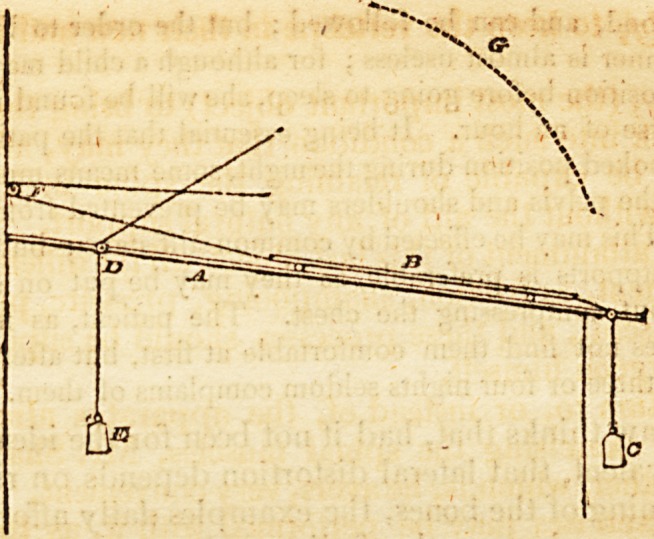# Mr. Shaw on Spinal Distortions

**Published:** 1824-03-01

**Authors:** 


### VIII.

*On the Nature and Treatment of the Distortions to which the Spine and the Bones of the Chest are subject. With an Enquiry into the Merits of the several Modes of Practice which have hitherto been followed in the Treatment of Distortions. Illustrated by Plates in folio.* By John Shaw, Surgeon, and Lecturer on Anatomy. Octavo, pp. 293; one Plate. London, 1823.

The world, as it now goes, and probably, as it has ever gone, is often more attentive to appearances than to realities—to the symmetry of the external form, than to the integrity of the internal functions. The mother, whose daughter's back deviates from the perpendicular, will fly to the charlatan who promises a speedy and certain restoration, rather than to the scientific surgeon who studies the mechanism, functions, and diseases of the spine. Such encouragement from the public has produced and fostered a host of regular quacks, and, perhaps, some quack regulars, in this department of the healing art; and, as might be expected, we have almost as many modes of treatment as practitioners on the spine. We are glad to find, that more than one or two surgeons of talent and knowledge are now prosecuting the pathology of the spine with zeal, as this is the most likely means of rescuing the diseases in question, from the hands of ignorance or knavery.* This class of disorders are of far more importance than is generally supposed. The spinal marrow exercises such an extensive range of influence over almost all the viscera of the chest, abdomen, and pelvis, that any encroachments on its form or structure must, more or less, impede or disturb their functions. For although the viscera will surprizingly accommodate themselves to distortions of the spinal column and ribs, as far as form or site is concerned, yet they cannot conform, with the same ease, to the interruption of nervous influence resulting from morbid changes in the column itself. We have daily opportunities of witnessing the derangements of function in the heart, lungs, and digestive organs, from diseases of the spine, and, therefore, recommend the study of these, not only to the surgeon, but to the physician and to the physiologist.

The present work is occupied solely with that kind of distortion termed "lateral curvature," Mr. Shaw reserving the other spinal affections for a second volume.

He properly observes, that it may be stated as a law of the animal economy, that the exercise of an organ is necessary not only to its perfection, but even to its preservation.

"In the investigation of this law, we are led to the curious and important conclusion, that the cellular membrane is not to be regarded merely as a modification of the original tissue or matter in which the peculiar substances that give character to *bone*, *muscle*, and *nerve* are deposited, but even that, from which other parts may be formed. The leading proofs upon which this opinion rests, are, that the several organs, unless kept in action, degenerate into cellular membrane; and that under certain circumstances, new organs, different in appearance and in function, may be formed, where nothing but cellular membrane was before visible." 2.

Thus, as long as a joint, for instance, is kept in activity, the apparatus continues perfect, and the various structures of which it is composed are abundantly distinct—but, when the motion of a joint has ceased for some time, all its complex parts degenerate into one homogeneous mass, resembling cel-

* We hope the prize offered by the London Medical Society for the best Practical Essay on Spinal Diseases, will call forth some valuable information on this subject.

lular membrane. The converse of these, Mr. Shaw observes, holds good. New organs, different in appearance and function, may be formed of cellular membrane. Thus, when the dislocated head of a bone lies for some time imbedded in the cellular membrane, cartilages, capsules, bursæ, ligaments, &c. are all formed from it, and this new joint, though it may not have the regularity of an original one, will perform the office tolerably well, the new capsule exhibiting all the characters of the proper synovial membrane. Various other examples are adduced of the functions and structures altering according to circumstances, shewing the plastic powers of Nature. But to return to the subject of degeneration of parts from want of exercise. The effects of inertion on the muscular structures is well-known, and too often exemplified, Mr. Shaw observes, in the wasting of the muscles of young people under a state of confinement for slight distortions, where they are encased in machines that not only prevent muscular exertion, but make pressure on, and cause absorption of, particular parts. The bones themselves waste away or become absorbed when no longer exercised, as is exemplified in the balls and sockets of dislocated joints, &c.

"If a soldier in active service receives a wound for which immediate amputation is necessary, or if the same operation be performed on a strong labourer while he is in full health and exercise, the bone is found hard as ivory, and compact in structure. But if either the soldier or the hospital patient should, in consequence of the accident, be confined to bed for some time before the leg is amputated, the bone is found soft and spongy, like that of a scrophulous person. A most remarkable instance of this is preserved in Mr. Bell's museum. It is part of the thigh-bone of a very strong man, who was a country brewer's drayman. The bone had been fractured, but, being badly managed, had never united. After the lapse of two years the limb was amputated; but the bone, though still of its original diameter, is not thicker in its walls than the tenth of an inch, the cavity having been filled up with a cellular structure, loaded with fat. A still better example is recorded by Cheselden, the most eminent surgeon and anatomist of his day. In his splendid work on the Bones, we find, in the explanation of the fiftieth plate, the following description:—'The thigh-bone of a soldier, who was shot in the right groin at the siege of Gibraltar, who being brought home the next winter, died soon after of a dropsy; the thigh-bone was wasted so much, as appears in the draught, and being weighed, after they were both sawed length-ways with a fine saw, the right weighed less than half the weight of the other.'" 9.

The same law extends to ligaments, nerves, arteries, &c. with some modifications in particular structures.

No part of the body offers more striking exemplifications of this law than the spine. The muscles that support the vertebræ become so weakened for want of exercise, as to be incapable of performing their functions. The vertebræ, and the ligaments which bind the vertebræ together, yield to the superincumbent weight;—and hence muscular weakness, though not the only, is one of the most frequent causes, or forerunners of spinal distortion.

The effects of general and regular exercise of the muscles are not more remarkable in producing symmetry, than the partial and undue action of them is, in producing disproportionate development of particular muscles, and in that way a species of deformity. This is seen in opera dancers, whose lower extremities are far more muscular than the other parts of the body; while in gold-beaters and blacksmiths it is the reverse. There is in exercise, as in every thing else, a golden mean, from which we cannot swerve far without danger.

"The capacity of yielding to a stretching power is natural to parts formed of cellular membrane, and possessing vascularity and life; but if this be done beyond a certain degree, and particularly if it be done suddenly or partially, a change of vascular action may be induced, attended with a visible alteration in the structure of the part. Thus, after a violent sprain, the ligaments become spongy and weak; or after long and undue stretching, they are unnaturally lengthened, and become elastic. This last effect is particularly evident in the lateral ligaments of the knees of chimney-sweeps, and in the condition of the ligaments of the feet of the opera-dancers. It may be observed, that the ligaments of the ankles of some of the most admired dancers are so unnaturally stretched, that in certain postures, as in the bolero dance, the tibia nearly touches the floor; so bad indeed is the effect occasionally produced by frequent stretching of the ligaments, that the feet of many of them are deformed; for the ligaments which bind the tarsal and metatarsal bones together, become so much lengthened by dancing and standing on the tips of the toes, that the natural arch of the foot is at last destroyed. This effect is very evident when the dancer is obliged to bring his heels to the ground, as in walking the streets; he then appears lame, the position having become almost unnatural to him.*" 18.

From the preceding and various other considerations Mr. Shaw thinks himself justified in coming to the conclusion that the most probable source of many distortions is either in "*the cessation of the actions of some particular part*, *or in the undue and partial exercise of others.*"

The occasional success attending the efforts of the charlatan, who is not guided by the light of anatomy or physio-

* "The gait of an opera-dancer, in walking, may be said to resemble, in some respects, that of a bear dancing; for this animal which, like all other quadrupeds, walks on the tips of his tops, when obliged to *dance* must bring his heel or os calcis to the ground."

logy, may depend often on his calling into action, by friction, &c. the dormant powers of Nature. The following case is offered as an example.

"A gentleman, having been thrown from his tilbury, hurt his shoulder severely, and a very eminent surgeon was sent for. Before he arrived, much swelling had taken place, and it was difficult to discover whether there was dislocation or fracture; in short, it was supposed to be only a severe bruise. As the patient, however, did not, in the course of two months, recover the use of his arm, he went to a rubber; who told him that his arm was dislocated, and proposed to reduce it. I was requested to see the gentleman, and found that the humerus was certainly dislocated, but in a very unusual manner, the head of the bone being laid on the infra spinatus space of the scapula. The dislocation could not have been directly occasioned by the fall, but consecutive upon the change produced in the joint by the bruise; for had such a displacement existed at the time when the arm was first examined by the surgeon, he could not have overlooked it, although the joint might have been very much swollen. The patient, however, thought otherwise: the surgeon was disgraced in his eyes; and the rubber having discovered a dislocation, gained his entire confidence. When I first saw this patient, he had no power over his arm; but by severe rubbing and pinching, while at the same time, the arm was kept in constant motion, the muscles acquired power; and a new socket being at length formed, the use of the arm was so far restored, that the gentleman was again enabled to drive. Although the operator was deceived in his expectation of putting the bone into its place, and although he was probably not aware that he had been acting on a correct physiological principle (for he had made a new joint,) yet we cannot deny that much good was done; nor can we be surprised that a patient, under such circumstances, should praise the quack at the expence of the surgeon." 22.

A knowledge of the principle on which success depends (observes Mr. Shaw) enables us to expose the tricks of those who pretend to push the bones of the spine into their places, after the vertebræ have (according to their language) been softened into a jelly by an hour's hard rubbing. The cases in which the benefit of friction, shampooing, pressure, thumbing, &c. (which are all modifications of exercise) is most apparent, are those of stiff and contracted joints, after rheumatism, or any chronic inflammation. But great perseverance is necessary in such cases—and a degree of boldness which, *à priori*, might seem dangerous—and sometimes is so. We find, however, that delicate patients bear much more severe treatment than could be expected; and the occasional success attending such bold and decided practice proves that, when judiciously applied, it may be a powerful restorative mean, particularly in contractions of the joints.

"Distortion of the spine is frequently the consequence of a disease which destroys the bodies of the vertebræ. During the active stage of this complaint, the rubber or machine-maker, unacquainted with the structure and diseases of the part, may, by operations intended to remove the distortion, so increase the irritation as to cause the death of the patient. But a very little knowledge will enable one, who by his conduct has acquired the opprobrious term *quack*, to understand that he cannot do so much as the surgeon can, during the active stage of the complaint. He therefore waits until the disease has been cured by anchylosis of the vertebræ (this, it is most necessary to state, is almost always attended with a degree of distortion which it is impossible to remove;) he then unblushingly makes promises, that in a certain time he will remove the deformity by restoring the bones, which he pretends are dislocated, into their proper places; being probably aware, that by rubbing and pressing the bones and muscles of a weakly patient, so much real and immediate benefit will be produed, as to give confidence in his promise of doing much more in time.

"It is generally said by such persons, that a certain time is required to soften the bones; and when that is done, it is easy to put them into their proper places. But it is more than probable, that those who can talk thus, are aware, that by rubbing and pressing the bones, they become harder; that they are not dislocated, but misshapen, by the destruction of part of their bodies (from which the distorted appearance of the whole spine arises;) that they are now firmly fixed and anchylosed; and that if it were possible to push them *in* or *out*, the operation would certainly be fatal to the patient." 29.

The next subject of enquiry is into the causes of the partial paralysis and wasting of one of the limbs during infancy, frequently producing distortion of the spine. In the great majority of these, observes our author, two circumstances seem to be common—"their connexion or dependence on the state of the bowels, and the effect they produce on the spine or limbs."

"The species of paralysis most interesting in relation to distortion of the spine, is that which is marked by a wasting or deficiency in the growth of a particular part, although unaccompanied with much defect, either in the power of sensation, or of motion.

"Such cases are not only important, in so far as they regard the organ affected, but in the influence which they have over other parts of the body; and this latter consequence is the more interesting, as by proper care, it may be counteracted.

"A diminution of size in one eye is the most common example we have of this affection; but neither this nor the diminutiveness of a finger, (unless for the deformity of their disproportionate size to the other organs) are of more consequence to the patient, than being indicative of the constitution being weak, and hence liable to more important defects. When one of the limbs becomes affected, not only is there a great deformity in the part itself, but the affection is often the source of distortion of the spine.

"Such an instance as the following is not uncommon:—A boy, on coming home from school during the holidays, was observed to limp in walking, and to appear crooked while standing. On being stripped, the left shoulder seemed to be lower than the right; but, on further examination, this effect was seen to be produced by the oblique position of the pelvis. On making him stand up, so as to bring the shoulders to the same level, the left heel was raised from the ground. And on carefully examining the left leg by measurements, it was found to be much smaller, in all its dimensions, than the right.

"In several cases nearly similar, the change in the character of the limb became evident, so soon after an attack of abdominal irritation, as to leave little doubt of the cause. But, in the greater number of such cases, the defect is seldom observed at the time of the first influence, and does not become evident until the other parts of the body are fully developed. In consequence of this, it is often difficult to trace these affections to their origin, and we are occasionally unable to determine whether they proceed directly from a certain condition of the bowels, or from a disease of the brain. It is probable, that in most instances, the remote cause is a deranged state of the bowels; the affection of the brain being as it were, intermediate between the disturbance of the bowels and the paralytic muscles." 34.

Mr. Shaw brings forward several cases and observations in illustration, for which we must refer to the work itself.

In respect to the treatment of these cases, it is evident that we must attend to the state of the digestive organs, and if actual paralysis have taken place, the local affection also must claim our attention. We must endeavour, on the principle that active exercise of an organ is necessary to its perfection of function, by friction, shampooing, warm and cold bathing, &c. to excite a certain degree of action. This, Mr. Shaw thinks, is to be assisted by mechanical contrivances to bring the paralysed muscles into play, and also to support them in certain positions while they are at rest. The particulars will be treated of farther on.

### On the Distortion called Lateral Curvature.

When the spine of a girl, about the age of fourteen, is becoming crooked, the attention of the mother is at first directed to the state of the shoulders or breasts—one either appearing larger than the other, or growing so unequally as to lead to a suspicion that it is diseased. In a still younger girl the state of the right shoulder attracts attention, as it appears larger than the left, and is often said to be "*growing out.*" The same is sometimes said of the hip.

"If the spine (which has probably been overlooked by the mother) be now examined, it will be found to be curved nearly in the form of the italic *f*. The whole of the right side will also have acquired a rounded and barrel-like form, while the left is diminished and contracted, the ribs being closer together than is natural. There is, moreover, a sinking in, or depression of the right, and a corresponding fulness of the left loin.

"This description nearly corresponds with the sketch of the girl's figure in the second plate given in illustration of the effects produced by a slight lateral distortion of the spine. The second figure in the same plate will assist us in forming a correct idea of the causes of the above changes in the form; for it will at once be evident, that the size of the right shoulder, the swelling of the same side of the neck, and the increased distance of the tip of the shoulder from the head, do not depend on any enlargement of the scapula or muscles of the shoulder, but on the change in the relative position of the ribs. It will also be obvious, that the appearance of the left hip being *out*, is caused by the left ilium being more elevated than the right, which is a consequence of the weight of the body being sustained principally by the right leg. The cause of the barrel-like form of the right side of the chest, and the contracted state of the left, is also obvious; and it is easy to comprehend how the projecting transverse processes of the lumbar vertebræ may give the appearance of fulness to the lower part of the same side." 50.

The cause of lateral curvature has given rise to much difference of opinion among medical men—some alleging that it depends on disease of the bones, while others maintain that it proceeds from disease of the muscles. Mr. Shaw, before examining into these opinions, endeavours to describe the manner in which the most common distortions take place.

While a girl, he observes, is in a slightly debilitated state, from fever, or from the change at a certain age, the lumbar portion of the spine often yields to the superincumbent weight of the chest and head—the yielding being at first so slight and gradual as to elude observation, until the upper part of the spine is curved in the opposite direction by the natural effort to restore the balance. Then the effects of the distortion become very remarkable, though the cause may still remain unobserved.

"If the spine were now to be examined, the case could hardly be mistaken; for in all probability, it would not only be twisted to one side, as the term lateral distortion implies, but three curves would be found in it. One (and which was probably the first formed) commencing at the pelvis, and passing towards the left side, as far perhaps as the tenth dorsal vertebra; from which the spine would probably take a gradual bend towards the right, up to the third or fourth; and from this, the upper part of the spine would take a slight curve towards the left, thus bringing the head nearly perpendicular to the pelvis." 53.

The above may be the general way in which curvatures take place in weakly children; but as they also occur in children who are robust, we must seek for some other causes than those described above. One cause Mr. Shaw traces to habits of standing or sitting in unnatural positions. Boys, in saying their lessons, often balance themselves on the left foot, the consequence of which is a slight curve of the whole spine. By long continuance of this habit a slight distortion is produced, and kept up or increased by mal-position while sitting writing, the right shoulder being kept more elevated than the left. The distortion will be still farther increased by using a soft feather bed with a high pillow, as he will naturally lie on the right side. If, in good health, the boy be permitted to run about and exercise all his muscles, he will soon recover from these slight distortions; but not so if he be neglected, if he fall into bad health—or enter a profession where he is much confined to one position. As may naturally be supposed, girls are more liable to these curvatures than boys, from their more sedentary habits.

"If a weakly girl of ten years old be obliged to sit for hours on a narrow bench, without any support to her back, it is not surprising, that notwithstanding all the reproofs she may receive, she endeavours to relieve herself by allowing the lumbar vertebræ to sink to one side. This may of itself be sufficient cause for the origin of a curve; but if the position in which girls generally sit while writing, drawing, playing the piano-forte, and more especially the harp, be taken into account with the causes already mentioned, it will be admitted that it is scarcely possible for a girl so situated to avoid being crooked, particularly if she is not permitted to take such exercises as give tone and strength to the muscles of the spine." 56.

Our author next endeavours to give a more particular description of the condition of the shoulders and ribs in common cases of lateral curvature, as their form alters very much according to the degree of distortion, or the plan of treatment pursued. Thus, when the spine is not much distorted, we shall probably find the right shoulder and breast larger and more prominent than the left—but if the curve of the spine be increased, the breast may be flat and contracted although the shoulder (right) be large and round.

"It may also be occasionally observed, that the breast and the upper part of the neck, on the left side, are prominent, while the same parts, on the right side, are lower, although the right shoulder-blade is more prominent than the left.

"That an increase of one side of the chest is the consequence of even a very slight lateral curvature of the spine, may be proved by bending the body to one side. This effect is demonstrated in the second figure of the second plate.

"But when the curve is increased, the ribs become flattened in front; and it is in consequence of this, that in general, the more prominent the shoulder is, the flatter the breast becomes. If the distortion be permitted to increase still farther, a complete change in the form takes place; the cause of this will be shewn in the explanation of the second and third figures of the third plate." 60.

For various other observations on the changes of form in these parts, we must refer to the volume itself.

Mr. Shaw now proceeds to examine the opinions which have been advanced and acted on respecting the causes of lateral curvature of the spine.

In London, at present (Mr. Shaw observes,) the idea is prevalent, that the distortion in question depends either on a diseased, or an undue and irregular action of the spinal muscles. But as the spine is almost invariably curved in two opposite directions, it is quite impossible to form an idea how the mass of muscles, running along the vertebral column could thus act in two different ways on the same side. If distortion depended on an undue action of the muscles of one side of the spine over those of the other, the curve should be always in the form of a single arch, instead of being serpentine, as it generally is. The practice formed on this theory in Germany, is to anoint the muscles on the contracted side with softening and relaxing unguents, such as goose grease, &c. whilst the weak muscles on the opposite side are to be rubbed with spirits and strengthening liniments. In England it has been proposed to keep the patient in the horizontal position until the equilibrium of the muscular power is obtained. The German practice, Mr. Shaw thinks, is at least innocent—but not so the English.

Another opinion has been advanced, that spinal distortions depend on a diseased state of the ligaments. But, although the form of the ligaments be altered in almost every case, the change, Mr. Shaw thinks, ought to be considered rather as the consequence than the cause of the disease; for a similar change may be produced by merely keeping the ligaments long in one position, as exemplified in the condition of the limbs in Opera-dancers and tumblers.

"In the spines of those who have been much distorted, I have found the intervertebral substance diminished on the concave part of the curve, in the same proportion as the bodies of the vertebræ; but I have never found any mark of disease in it; on the contrary, in the cases which I have examined, it has retained all its peculiar firmness and elasticity." 72.

"*Is lateral distortion ever consequent upon dislocation of the vertebræ?*" A distinct and peculiar mode of practice having been founded on the theory of dislocation of individual vertebræ being the cause of distortion, our author observes ;—

"There has been no example of actual displacement of one vertebra from another producing distortion, similar to those that may be daily seen, recorded by a person of any authority. The last, and certainly the best authority upon this question, Sir Astley Cooper, says, 'It has been generally stated by surgeons, that dislocations of the spinal column frequently occur. But if luxation of the spine ever does happen, it is an injury which is extremely rare; as in the numerous instances which I have seen of violence done to the spine, I have never witnessed a separation of one vertebra from another, through the intervertebral substance, without fracture of the articular processes; or if those processes remain unbroken, without a fracture through the bodies of the vertebræ. Still I would not be understood to deny the possibility of dislocation of the cervical vertebræ, as their articulatory processes are placed more obliquely than those of the other vertebræ; from the vicinity of our hospitals to the river, sailors are often brought into them with injuries of the spine, by falls from the yard-arm to the deck; and as there is almost always an opportunity of inspection in these cases, a dislocation must be extremely rare, since I have never met with a single instance of it, they having all proved to be fractures with displacement.'

"I may refer to the Appendix to shew that the instances of injuries of the spine, preserved in Mr. Bell's Museum, corroborate the opinions expressed by Sir Astley Cooper." 74.

Mr. Shaw has had, by accident, an opportunity of judging of the nature of the evidence which has been adduced in support of the opinion, that dislocation is a common cause of distortion, and in this place contradicts a statement made in the 44th volume of the Medical and Physical Journal, by Dr. Edward Harrison, respecting a child knocked down by a Hampstead coach. Dr. Harrison had stated as follows :—

"Upon examination I found the first lumbar vertebra wholly dislocated and driven into the left loin. The right transverse process is sunk downwards; the opposite one has risen, and can be distinctly felt below the skin. The last dorsal and second lumbar vertebræ were also displaced by the accident. Several other dorsal bones are also suffering spontaneous luxations from the want of vertebral support below them." *Loco citato.* 77.

Mr. Shaw, on the other hand, could not discover any of the dislocations above-described, when examining the child, who died suddenly of croup. The note he made at the time is as follows :—

"There is a fracture in a horizontal line in the middle of the first lumbar vertebra, the lower half continuing attached to the other lumbar vertebræ, the upper to the dorsal. The two portions are united together obliquely by a ligamentous matter, so that, at this part, there is an appearance of dislocation; but there is not the slightest displacement of any of the other vertebræ. The spinal marrow is completely destroyed at the fractured part." 77.

Several severe strictures are here passed on Dr. Harrison's practice, which we conceive he is bound to answer, and therefore shall not dwell on them in this place.

The next enquiry is into the opinion, that lateral distortion is caused by disease of vertebræ. This opinion is embraced, Mr. Shaw observes, by many of the best informed members of the profession. Our author does not deny that disease of the spine will produce distortion—but he thinks that the distortions, in these instances, will generally be found very different from lateral curvature, being either consequent upon caries of the vertebræ, or upon *ricketts*, a disease that especially attacks the osseous system.

The *post mortem* reports of cases of lateral curvature generally represent the bodies of the vertebræ, as softened and full of a scrofulous substance; but this, Mr. Shaw thinks, is incorrect, for in the greater number of cases, the internal structure of the bodies of the vertebræ has a natural appearance.

"It is easy to account for the mistake. If the vertebræ of a patient, who has long been confined to bed, be examined, the appearance described above is found; but if the person has been in the habit of taking exercise a short time previous to death, the bodies of the vertebræ are discovered to be as firm and compact as those in a perfect spine. I trust that it is now almost unnecessary to add, that instead of admitting that the softened condition of the vertebræ affords a proof of the existence of a scrofulous disease, that it should be considered as merely shewing the bad consequences of confinement and want of use. The wasted appearance of the muscles, which is occasionally observed in cases of lateral curvature, and which has been confounded with their state during the inflammatory stage of the carious diseases of the spine, also depends on the same causes as the softening of the bones.

"It is well known that the shape of the vertebræ is materially altered in cases where the spine is much distorted; but as no mark of disease is discovered when a section of the bones so mis-shapen is made, we may infer that the change of form is a consequence that may be produced independent of any specific disease existing in the bones, especially as it is found to correspond to the direction in which the pressure has been made." 95.

In fine, Mr. Shaw comes to the conclusion that, with few exceptions, the bodies of the vertebræ, in lateral curvature, are not affected by any specific disease—and that they are not so liable as the more solid bones to be inflamed by the irritation of pressure.

In Mr. Shaw's experience, there have not been that pain in the back, and wasting of the muscles which are generally stated as accompanying lateral curvature. There is, however, occasionally, a weary pain in the back, which, in slight distortions, he attributes to weakness, as it is quickly relieved by certain exercises which would increase rather than diminish pain consequent on inflammation or disease of the vertebræ.

Mr. Shaw next describes the plates, introducing anatomical and pathological remarks, which cannot be understood without reference to these plates. From these descriptions, however, we gather that our author considers the distortions attending lateral curvatures as generally depending not on disease or material alteration in the structure of the bones, but simply on *curvature* of the spinal column. Another conclusion to which our author comes is, that in lateral curvatures, the pelvis is never distorted by the application of instruments, as had been very generally suspected, especially since the lectures and writings of Mr. Wilson. The following extract will convey some idea of Mr. Shaw's opinions respecting curvatures of the spine, with or without disease of the bones.

"The specimen represented in the fourth plate is offered as an example of the extent to which the spine may be distorted, independently of any specific disease of the osseous system. The proofs upon which this opinion rests were first drawn from a comparison of a number of distorted skeletons. It was often found, that although the spine and ribs were distorted to the greatest possible degree (as in the specimen before us), there was no deformity of the limbs; while on the other hand, the limbs were often much distorted, although the spine was little affected. From an observation of these facts, I was led to conclude, that among the great varieties of lateral distortions, there were two distinct classes, differing most essentially from each other. On farther investigation it was found that in the one, the distortion seemed to be owing, or at least to accompany a specific disease of the osseous system, which evinced itself in some of the long or solid bones; while in the other, no marks of disease were found, and the distortion was confined to the small bones which are bound together by ligaments, and are dependant on a muscular apparatus for the preservation of their form.

"From these facts, I drew two conclusions, which, if correct, will be acknowledged to be of some importance in practice. 1st, That unless some of the long bones, or the bones of the head, be affected, however distorted the spine may be, we are not to consider the disease as ricketts, i. e. that the distortion does not depend on a specific disease of the bones; and 2d, That the spongy bones (as of the spine, carpus and tarsus) are not so liable to be affected by ricketts as the long bones and those which are more compact in their structure." 126.

*State of the Pelvis in Lateral Distortions.* This is a subject of considerable importance, and can only be decided by the testimony of experienced practitioners. In several of the plates where there are distortions of the spine there are no distortions of the pelvic bones. In some, however, these last are seen distorted, but in all of these, there is evidence of ricketts. "So far we have proofs," says Mr. Shaw, "that there are many cases, in which, although the spine be distorted, the pelvis is not affected."

"But I shall even venture to say, that on prosecuting the enquiry further, we shall be induced to conclude, that in whatever state of distortion the spine and ribs may be, the bones of the pelvis will not be found distorted, unless there be at the same time, marks of ricketts in some of the long or solid bones." 128.

In addition to the series of distorted pelvis, in Mr. Bell's collection, which corroborate Mr. Shaw's opinion, he offers testimony to the same effect from the writings of Sandifort, Cheselden, and Walther.

"The above opinions have been, in a great measure, proved to be correct, by the concurring testimony of the most eminent accoucheurs in London. Several of these gentlemen have told me, that it was not unusual for ladies who had distortion of the spine to have easy and natural deliveries, although they had been sent up to town for their first accouchement, under the idea that there would be a necessity for the assistance of instruments; while, on the other hand, the pelvis was often so deformed, in cases where there was no curvature of the spine, as to require the aid of instruments.

"I was much gratified to find, that the mark which I had been led to consider as affording the best criterion, by which we may judge of the probability of the pelvis being distorted, corresponded with the experience of the same gentlemen; viz. the condition of the thigh bones, or tibiæ. Having been lately in Edinburgh, I had an opportunity of conversing with Professor Hamilton on this question. He told me that he had frequently met with instances similar to those mentioned in the preceding page. He moreover made an observation which I consider highly valuable, as affording another mark, by which we may discover the condition of the pelvis:—He seldom or never found the pelvis affected, unless the distortion of the spine had commenced in early infancy. To those acquainted with the disease of ricketts, the value of this remark will be obvious; for they must recollect that it is generally between two and five years of age that the first effects of ricketts upon the bones are observed. It is scarcely necessary to state, that the common lateral distortion seldom takes place until a child is nine or ten years old." 132.

In respect to the liability of the bones of the pelvis to be distorted by the application of instruments, Mr. Shaw is bold enough to assert that, unless there be a specific disease of the osseous system shewing itself in other bones besides those of the spine, we need not be under any apprehension of distorting the pelvic bones by the said application of instruments. After quoting Mr. Wilson's sentiments on this subject, which are well known, Mr. Shaw observes that the pelves to which Mr. W. alluded were chiefly from Mr. Bell's museum, and those of the College. Both of these collections Mr. Shaw examined, and says they were examples of distortion from ricketts, which disease is well ascertained to distort the bones of the pelvis, and often in such a manner as to resemble the compression from machinery, where no instruments had been used. Mr. Wilson's mistake arose, Mr. Shaw thinks, from confounding lateral distortion consequent upon weakness with that dependant upon, or accompanied by, a specific disease of the bones.

The next subject discussed, is that of the effects produced on the viscera by distortion of the bones of the trunk.

When we contemplate the skeletons of distorted subjects, it is evident that the viscera must have been very much encroached upon and altered in shape, though not so often in texture as we might expect. But although we must grant, with Mr. Shaw, that we every day see distorted people attain old age, we are not inclined, from thence, to infer, that organs incommoded and altered in position, as those of deformed people generally are, "still perform their functions properly." Mr. Shaw, however, only means that this is sometimes the case, for he immediately after adverts to the functional diseases consequent on distortion of the spine. The heart and lungs are the organs principally affected by its function in these cases—or rather we should say the organs of digestion, which we have very frequently seen deranged from spinal disorders. Symptoms of organic diseases of the heart are not unusual in these cases, and are with difficulty distinguished from real affections of structure in that organ. When we have distortion, however, we may generally give a more favourable prognosis, as the organs in time accommodate themselves to the encroachments of the surrounding parts.

"I had, at the same time, two patients who were supposed to be dying of disease of the heart. The one was a young lady who certainly had all the symptoms of disease of the heart, for she had great palpitations, with an irregular pulse, and she used frequently to faint; but these symptoms came on after she had been long confined nearly in a horizontal posture on the inclined plane, without being permitted to take the slightest exercise. Such a debilitating plan of treatment was sufficient to account for the source of most of the symptoms; and on examining the condition of the spine, it was easy to explain the cause of the palpitations of the heart being more distinct than usual, for the spine was so twisted, that the heart was carried forwards, and its apex seemed to pass between the ribs. That this was the true explanation was easily proved, as there happened to be at the same time, another young lady under my care, in the same house, whose spine was curved in the opposite direction. In her, the natural pulsation of the heart could not be felt on the left side." 144.

The effects of these distortions on the lungs are considered by Mr. Shaw as comparatively of very little importance. In many cases which he has examined, where the lungs were affected, it has appeared to him, that the malformation of the chest was a *consequence* of disease of the lungs at an early period of life. "This opinion is founded on the belief that there is the same correspondence between the lungs and their external apparatus, viz. the bones and muscles of the chest, as there is between the brain and the scull." Laennec has given many cases where diseases of the lungs has produced alterations in the form of the chest.

"Although it may be doubted whether distortion actually produces disease of the lungs, or whether a disease of the internal organ can be the cause of an alteration in the form of the chest, after a certain age, there can be no question of the practical benefit to be derived in considering the lungs and the chest as mutually dependant. The almost immediate good effect which results from a patient, who is nearly on the verge of consumption, regularly going through exercises, by which the muscles of respiration especially are brought into action, is so obvious, that the most sceptical theorist must, on observing it, be convinced of the close connexion that exists between the external and internal organs." 149.

*Treatment.* We now come to the enquiry respecting the several modes of treatment, and first of "*friction, thumbing*, and *shampooing*." On these Mr. Shaw makes but a very few observations. Although much mischief occasionally results from the attempts made by rubbers, who cannot distinguish between a distorted joint, in which a specific disease is still dormant, and a lameness consequent merely on the contraction produced by inflammation, still we must admit that wonderful changes are often produced by each of the processes abovementioned, not only in relieving stiffness and contraction, but even in restoring strength and vigour to weakly persons with slight distortions of the spine. These good effects would be still greater, of course, were the means under the guidance of people acquainted with anatomy and pathology.

*On the Reduction of Vertebræ said to be Dislocated.* After what has already been said on this subject, Mr. Shaw thinks it unnecessary to offer fresh facts in proof of the fallacy of the opinion, that the situation of the individual vertebræ, in a distorted spine, can be changed.

But he considers it incumbent on him to endeavour to explain the cause of the degree of success which occasionally results from the mode of practice followed in the attempts to change the places of the vertebræ. The process, he observes, has been kept as secret as possible, and, therefore, cannot be fully canvassed; but from the descriptions given by those who have witnessed the operation, we may conclude that its occasional good effects result from the confinement of the patient to the horizontal position, while the muscles of the spine are, at the same time, kept in such a state of activity by friction and pressure, as to be prevented from wasting. In this way, the bad effects of close confinement to the same position are, to a certain degree, obviated.

In respect to the last mentioned practice (confinement on a horizontal or inclined plane for months or years) the arguments that have been offered in favour of the plan are founded on the idea that the distortion depends on an undue contraction of the spinal muscles, and a diseased state of the vertebræ.

"It is scarcely necessary to repeat the arguments that have been already offered in refutation of these views, nor are proofs now required to shew that every part of the body is weakened and deteriorated by lying dormant. But I may state, that it is scarcely possible to imagine any means so effectual, in preventing parts from performing their natural functions, as the plan proposed for the cure of a disease originally proceeding from weakness. Indeed, this now begins to be discovered, and the use of the inclined plane is gradually falling into disrepute. For it is found, that although a girl who is slightly distorted may become more straight after having been confined to the horizontal position for months, she does not gain strength, but, on the contrary, becomes so weak, that she can scarcely walk or stand; and when she attempts to sit up without some artificial support, she sinks almost double, or at least into a state worse than she was in when she first lay down. These are sufficient objections to the practice, but the effects which a long continuance in the system has upon the general health, are still more serious. We find that girls who have been long confined to the reclining board are delicate and liable to all the worst symptoms of hysteria; and I have already mentioned an instance of a young lady having so many symptoms of diseased heart, that she was treated accordingly, although they were afterwards proved to have proceeded from the weakness caused by long confinement to one position." 158.

In making the observations, our author does not mean that the inclined plane should be entirely abandoned. On the contrary, it forms an essential part of the plan of treatment he proposes for lateral curvature. Nay, he would even recommend that a delicate girl, although she may not be in the slightest degree distorted, should lie for some time every day upon the plane. It is true that the spine of the most delicate girl will not suffer by the upright position, *while she continues in a state of activity or exertion*—it is during a state of lassitude and relaxation of the muscles, that the horizontal posture is necessary, as it is then that the ligaments and bones yield.

*Machines*. Many and weighty objections are made by Mr. Shaw to the application of instruments for stretching and supporting the spine. Mr. Chesher, of Hinckly, is supposed to make use of this means only, by which he has gained great celebrity. But Mr. Shaw has reason to think that Mr. Chesher's method is either misrepresented or misunderstood—his remarks, therefore, apply to the general principle of mechanical extension, without reference in particular to Mr. Chesher's plans.

The objects proposed to be obtained by the application of what is called Mr. Chesher's collar, and certain improvements on it, are—to stretch the spine when it is curved—to keep it stretched—and, finally, to remove the cause or source of the distortion.

"The machine is fixed firmly on the pelvis; and when the body is elevated, by pulling upon the head-piece with the cord and pulley (the straps being under the chin and the back of the head), the upright bar is fixed. As there is no elasticity in this bar, nor any provision for its being elevated or depressed, in correspondence with the motions of the body, the head must be invariably kept at the same distance from the pelvis; and as the head was forcibly elevated before the application of the instrument, great part of the body must be hung as an appendage to it, the whole weight being supported by the straps under the chin and the back of the head.

Independently of the objections that may be made to the pain which necessarily attends the operation of the instrument, when applied in the manner supposed to be necessary to stretch the spine, it may be stated, that there is no longer any exercise of those muscles by the action of which the spine is supported in a state of health; and, consequently, they become gradually weaker, and every day less able to support the spine.

As a natural result of this, we find, after the instrument has been worn for some time, that either it, or a machine similar in effect, is indispensably necessary to the patient's comfort, unless the spine has become a solid and immoveable column, by the process of anchylosis." 164.

The objection, that these machines distort the pelvic bones is not well founded, in Mr. Shaw's opinion. But they certainly have some effect in producing distortions or changes of form in the face. The grand question is, the curability of disease by wearing such machines. The only answer Mr. Shaw can give is this—that he could offer instances of persons who are still wearing the collar, although it is more than twelve years since its first application—and of others who have given it up, and are now more distorted than they were before. Still these instances of failure do not disprove the occasional utility of the machine. Certain cases of disease of the vertebræ may, he thinks, be benefitted by the collar, and in the chapter on treatment, Mr. Shaw himself proposes an instrument on the same principle, but more simple, as necessary to the cure of lateral curvature.

In respect to the *stretching chair*, Mr. Shaw considers it as not entirely devoid of danger; for the windlas by which the crane is elevated, and to which the patient's head is attached, is so powerful, that it might almost tear the head from the body.

"This apparatus is occasionally used, but not so frequently as the rope and compound pulley, which is also so powerful that a grown-up person can be easily raised from the ground by it. This is used not only to raise patients from the ground, but to suspend them for some time. Until I saw several patients undergo this experiment, I could not believe that it was ever put into practice, for it is quite obvious, that while a child is suspended by the chin, the ligaments of the neck must be stretched to a dangerous degree, while the lumbar portion of the spine, where the principal curve generally is, is scarcely affected." 175.

Still the plan of stretching the spine, under certain regulations, is not objected to by Mr. Shaw—on the contrary, one of his own measures for curing distortion rests on this process.

The plan of carrying a weight on the head, as proposed by the late Mr. Wilson, is considered by our author as a useful adjunct in slight cases, but not effectual by itself in curing distortion. If a light weight be used, scarcely any harm can ensue, "but if even a few pounds be not carefully adjusted on a particular part of the head, the sterno-cleido mastoidei muscles, and others on the fore-part of the neck are liable to be brought into too much action. If too great weight be placed on the head there is danger, he avers, not only of the lumbar part of the spine being carried inwards and bent, but even of the pelvis being diminished, in consequence of the two last lumbar vertebræ and the upper part of the sacrum being driven inwards. It appears indeed that Andry, Professor and Dean of the Faculty of Physic of Paris, (1723) lays down, in a little work entitled *Orthopœdia*, directions for this mode of curing distortions. He advises, that a powder-box or other light article be placed on the child's or person's head, and that he be directed to walk about, taking care not to let the article fall off; by which process the spinal muscles will be daily exercised.

*Exercise.* No single method, our author observes, of treatment is so effectual in counteracting or curing slight distortions of the spine, as properly regulated exercise. The indiscriminate use of violent exercise, however, in every kind of distortion, must be productive of much mischief. In anchylosed spine, the part which is ossified is far more liable to fracture than that which is not anchylosed.

"When there is only a degree of lassitude, and before distortion has actually taken place, a variety of exercises are safe and useful; but when the spine has become in the slightest degree distorted, it is necessary to pay strict attention to the effects produced* by each kind of exercise." 187.

We now come to Mr. Shaw's own principles of treatment; but fear we shall not be able to give any thing like a clear idea of this part of the work, on account of the numerous diagrams introduced to elucidate the mechanical exercises proposed or recommended by our ingenious author. He observes, and not without reason, that "the modes of practice for the cure of distortions should be as numerous and as varied as the causes upon which the different cases depend." But this is not all. "In each case there are so many distinct stages, that during the progress of the treatment it is sometimes necessary to use means which appear opposed to each other in principle." This is sufficiently discouraging; but Mr. Shaw hopes that the pathology of the disease has been so enumerated as to enable the reader to comprehend the principles upon which the modes of practice proposed are founded.

Our author remarks that, if a lateral curvature of the spine be observed at its commencement, it may easily be cured; but parents too often neglect the distortion or take improper means to remove it, till it has proceeded to a considerable extent.

"For example, if one shoulder projects rather more than the

---

"* It is by attention to this, that Mr. Jenkins, the celebrated teacher of dancing, has been so successful in preventing, and even in remedying, slight degrees of distortion."

other, or if one side seems a little larger than the other, a pair of stiff stays and a collar to brace the shoulders back are immediately applied; and this plan is persevered in, for every person in the family is delighted to see how much the child's figure is improved. But although the evil may be concealed for a while, its cause is increased by wearing stays or a collar, for the child can no longer take that sort of exercise which is necessary to keep the muscles of the spine in such a state of activity, as to fit them for their several uses." 191.

It has been remarked by old and intelligent people, that deformity is much more common now than thirty years ago; and the reason they assign is, that it proceeds from too much care being taken to preserve the shape. This our author believes to be partly true.

"But this is not the only cause; for it must be admitted, that more attention is now paid to the various accomplishments of writing, drawing, and music than formerly; and consequently girls are more liable to be kept in positions which are prejudicial to the shape. If a girl is naturally strong, and is permitted to have enough of active exercise, she may counteract the ill effects of long continuance in a bad position; but if she be weakly, and have not proper exercise, the lower part of the spine must yield to the weight of the upper part of the body. Taking this view of the causes of distortion, I would concur in part with the opinion of those who believe that stays are useful: however, they should be worn only by weakly children to prevent the spine from sinking while they are obliged to sit up; for stays will never cure a distortion, nor give strength to the muscles. We have only to observe the fine figures of the peasant girls, to be convinced that stays are not absolutely necessary; but if children are brought up artificially, they must have some artificial support." 197.

Our readers are aware of the remarks which Mr. Shaw has made on the bad effects of mal-position and sedentary habits among weakly young ladies, and these should be counteracted as speedily as possible by the medical attendant. After commenting on many of the injudicious or injurious means which have been invented to keep the head back in those who are inclined to stoop, our author introduces an illustration which we think contains much ingenuity and good reasoning. We shall give an extract from the work containing this illustration.

"However, although the collars and the lead weight, as they are generally used at present, are not only inefficacious but even hurtful, they may be of considerable use in keeping the shoulders in a certain position, after they have been brought to it by such exercises as tend to strengthen those muscles of the back by which the shoulders and head are naturally supported. But so completely do

I differ from the opinions commonly entertained, as to the means of counteracting an habitual stoop, that I would almost recommend the position of a tailor sitting on his shop-board, as more advantageous than the systems generally followed. This at first appears ridiculous; but the manner in which a tailor holds his body when he walks, proves that there is something in his habits which tends to the correction of a stoop; for he is quite a caricature of a strutting erect figure, especially in the way he bends in his loins and carries his head.

"The peculiarity of the tailor's gait depends in a certain degree on the bent position in which he sits; but this explanation is not at first satisfactory, since it may be observed that other tradesmen, who also stoop while at work, generally have their head inclined forwards, and have also a distinct and habitual bend in the neck; such, especially, may be seen in persons who sit at the table and stoop forwards, as watchmakers, engravers, &c. It is not difficult to explain the cause of the difference, and the enquiry will assist in directing us to the principles which we ought to recollect in our operations upon the spine.

"In the sitting position of the tailor, the head hangs so low, and so complete an arch is formed between it and the pelvis, that the muscles of the spine are called into strong action to support the head; the necessary consequence of this is, that these muscles become even unnaturally strong, or at least so as to predominate over those by which the spine is pulled forward. But the bent position is not the only cause of increase in the strength of the mucles, for it depends also on the exercise given by frequently jerking the head backwards. In those who stoop from the middle of the body, as in writing or working at a table, the muscles of the spine are not called into similar action; for while the head is in this position, it rests or is supported by the ligamentum nuchæ. The ligament being thus kept constantly on the stretch, becomes lengthened; and hence the stoop is increased. When this is combined with the consequences of the want of muscular action, the deeper ligaments which bind the cervical vertebræ gradually yield; if the operation of these causes continues for a certain time, the vertebræ themselves become altered in shape, and consequently an almost irremediable stoop is produced.

"These ideas are corroborated by what may be observed in the shape of the tailors in some parts of Germany, who instead of having the erect figures of London tailors, are quite bent. On enquiring into the cause, we find, that instead of their sitting as tailors do in this country, a hole is cut in the table, and a seat is placed within it; so that their position, while working, becomes nearly the same as that of persons who sit at a table." 209.

Although it would be ridiculous to propose the position of a tailor to a young lady with stoop-shoulders, yet Mr. Shaw has found it useful, in very young patients, to put all their play-things on the ground, recommending them such games as may induce them, while sitting, to bend the body and raise the head alternately. In patients farther advanced, much benefit has been derived from the use of an instrument which was planned for bringing the muscles of the spine into action. We shall give the description and plans of this apparatus, as we find them in the work.

" There is a sketch of the application of the apparatus in the seventh plate. It consists of an upright rod, four or five feet high, and similar to the pole of a common fire-screen. In the upper part of the rod, a small wheel is placed, and about sixteen inches below the wheel, a lever eleven inches long is *let in*, and moves upon a pin; the opening in the rod being so cut as to permit the lever to rise and fall. To the end of the lever, a certain weight is attached. This apparatus may be fixed by a small wooden vice to the table, directly opposite to the girl as she sits at her lesson, or at work. A riband, with a loop or ring in front, is to be put round her head; a silk cord is to be attached to the loop, and is then to be carried over the wheel in the rod, and to be fixed to the end of the lever, the cord being so adapted that when the girl is sitting quite erect, the lever is raised as high as possible. As long as she sits erect, the lever being kept up, there will scarcely be any weight dragging upon the head; but if she stoops or lets the spine fall to one side, then the cord is relaxed and the lever falls; and by the consequent increase of the power of the weight, the force becomes considerable. To relieve herself from the constant pull, she is obliged to sit upright; and by the exertion to counteract the force of the weight when it falls low on the scale, the muscles by which the spine is naturally kept erect, are much strengthened. This instrument is very manageable; by moving the cord back upon the lever, the force or weight will be much increased, while by keeping the cord attached to the end of the lever, and moving back the weight, the force will be diminished. The adjoining plans will assist in the demonstration of the uses of the apparatus.

Fig. 3 Fig. 4

"The cord A passes over the pulley B, and is attached at C to the end of the lever E, which moves upon a pin in the upright rod at D. To the end of the lever the weight F is hung. It is evident that as long as the lever is in the position of fig. 1. there will be little difficulty in supporting the weight by pulling upon the cord; but as the lever falls to the position in fig. 2. the force required will be in proportion to the distance it reaches down the scale. Without increasing the size of the weight, the force may be greatly augmented by moving the cord back upon the lever, as in fig. 4; and if we wish to diminish the force, it is only necessary to change the place of the weight, as in fig. 3." 214.

Some other apparatus are described by our author; but the principle is to give the muscles of the spine alternate exercise and repose. Balancing a light weight on the head is useful. As there are generally several curves in the spine, in cases of distortion, care must be taken that the exercise which is useful in counteracting one, may not have the effect of increasing another. Any person, however, who is at all conversant with the mechanism of the spine, must be able to judge of the proper exercises in such cases. One kind of exercise may be useful in all distortions, and can be injurious to none—"*it is the act of bowing the head as low as possible, and rising slowly with an exertion*"* It is very easy to employ an apparatus that may increase the effect of this mode of exercise.

"Previous to describing the means that are necessary to cure a confirmed twist, I may state, that although, in slight cases, properly regulated exercises are very beneficial, still comparatively little good will be derived from them, unless strict attention be at the same time

---

* Does the common usage of the above practice among our *northern* neighbours in this country conduce to the straightness of their spines?—*Rev.*

paid to the patient's manner of sitting and standing. Particular care ought also to be taken that she should not lie crooked in bed, and that she should use a mattress instead of a feather-bed, with the head as little elevated as possible. Although the practice followed of confining patients with slight distortion to the inclined plane for months, without intermission, has been shown to be bad, yet, lying for a certain time on the plane, and especially after having gone through the proper exercises, is of the greatest use to those who are slightly distorted. With reference to the question of the propriety of rubbing, or shampooing, I would say that it is a species of exercise, of which a patient cannot have too much." 221.

*Treatment of confirmed Lateral Distortion.* Mr. Shaw commences this chapter with some useful directions respecting the examination of spinal distortion, with the view of ascertaining whether it depends on a specific disease of the bones, or on general constitutional debility. We should, for instance, enquire whether the patient suffered any severe illness during infancy? What was the condition of her first set of teeth? Whether she had ever been liable to swellings in the neck? At what age the distortion was discovered, &c. We are next to examine the form of the head and face, and whether the thighs and bones of the leg are perfectly straight.

"In examining the spine, we should request the patient to stand in the position she is most accustomed to; and, after looking at the general form of the spine and chest, we should mark the situations of the spinous processes of the several vertebræ, by dotting the skin over them with ink or rouge, at intervals of about an inch.* The patient ought then to raise herself with an exertion, and while she stands fairly on both feet, and as erect as she can, we should observe whether the positions of the spots, by which the situations of the vertebræ were marked, are altered. We may now request her to stoop forward, and to bend the spine as much as she can. While she stands so, we may, by rubbing firmly with the point of the finger upon the spinous processes, make a red line along the course of the spine. This may be the first thing done, or the apices of the spinous processes may be dotted with ink, while the body is bent as much as possible; the intention in marking the back, being not only to shew the exact situations of the several vertebræ, but also to assist us in ascertaining whether the curves can be affected by a change of position.

"If the spine be made straight by the patient endeavouring to stand erect, or if the lateral curves are lessened by the bent position, we may consider the case favourable.

"We should not omit to enquire whether any pain is felt, when

---

"* If the plumb-line is used in the manner described at page 112, a correct idea of the deviations of the spine may be easily formed."

particular vertebræ are pressed upon. If the patient complains, (which on the first examination she is very apt to do,) we should try whether rubbing on any other prominent bone is equally painful.

"By the above manner of conducting the examination, we may gain nearly an accurate idea of the state of the vertebræ; but the characters of the curves will be more satisfactorily ascertained by elevating the head so as to stretch the spine. If the patient is very young, a servant standing on a chair may raise her by the chin and back of the head; but it is more convenient to use a double pulley, which by a simple contrivance, may be temporarily fixed to the top of a door.

"The patient should sit on a chair, and the cord being fixed to soft straps under the chin and back of the head, is to be steadily pulled (by the surgeon,) so as gradually to elevate the head. This should be done with great nicety and gentleness, as there may possibly be anchylosis between some of the vertebræ. The symptoms of inflammation or caries of the spine are so distinct, that no surgeon could mistake them for those of lateral distortion; and consequently would never propose to examine a spine so affected, in this manner." 225.

If the patient be laid at full length on a plane surface, and the position of the heels compared, one leg will *appear* longer than the other—the apparent difference being a consequence of the position into which the pelvis is thrown by the curve of the lumbar vertebræ. A patient with distorted spine generally walks in a peculiar manner, one side being carried rather before the other.

"In considering the possibility of curing a distorted spine, it may be stated, that if the patient is young, and if there be no anchylosis, nor any symptoms of ricketts or disease of the bones; if the cervical or upper dorsal vertebræ are not so much distorted as to form a large swelling on one side of the neck, and if the middle of the ribs are not yet become angular, we may safely promise to make the patient much better; but in justice to ourselves, we ought to state, that all our attempts will be ineffectual, unless the patient assists by submitting cheerfully to the rules laid down. A girl, even so young as thirteen, with a distorted spine, has no time to lose, and if she expects to have her shape restored, she must consider the attempt to remedy it, as her sole object for many months.

"There is one argument in favour of making the attempt as early as possible, and the correctness of which is established by every day's experience;—after a distortion has proceeded to a certain extent, it becomes rapidly worse, if neglected or mismanaged; and the longer it continues, the less susceptible it is of remedy." 230.

The objects which our author proposes to attain by combining the various remedial agents, are the following:—

*First*, to act upon the spine so as to alter the present position of the vertebræ, and consequently of the ribs and shoulders.

*Secondly*, to keep the vertebræ in their new and improved position.

*Thirdly*, (the most important object) to bring the muscles of the back into such a condition that they may, after a certain time, be capable of retaining the spine in its natural position, without the aid of any natural support.

For the attainment of this last object, all the different means of excitement, as friction, shampooing, pressure, percussion, &c. are useful, and these processes should be superintended by the surgeon himself.

In addition to, or instead of, the apparatus already described for exercising the spinal muscles, Mr. Shaw recommends another, which is, he avers, very convenient. "Indeed so many exercises may be performed with it, as to make it useful in almost every stage of distortion, and it, at the same time, forms a better reclining board than those generally used. We shall here introduce two sketches of the apparatus recommended by Mr. Shaw.

Upon the frame of a sofa the board A is placed. It is now horizontal, but may be made an inclined plane by raising the end to C or D. The patient should stand at the upper end of the sofa, and lean over it. With a band fixed to the head, she should raise the weight E, until her body becomes erect; she is then to bend the head, so as nearly to touch the board, and to go on for some time, alternately rising and stooping.

As it is difficult to induce patients to persevere in the performance of one exercise, Mr. Shaw has endeavoured to contrive a variety of them to effect the same object. By means of an additional board to the plane (which is absolutely necessary to it as a stretching or reclining board) a number of useful and amusing exercises may be performed.

"No. 2. is the plan of the most simple form. Upon the plane A, which is considerably elevated, there is the moveable board B. If the patient sits upon this, and pulls upon a piece of wood attached to a cord, which, after running through a pulley, F, is fixed to the board B, she will draw up the board; and if the arms be at the same time kept at full length, the body must fall back in the direction of the dotted line G, thus acting upon the vertebræ of the loins. The power required to raise the board, will depend on the degree of the elevation of the plane, and on the weight C. This exercise is very good for the curve in the loins, but it produces little effect on that, in the upper part of the spine. This, however, is easily effected, by putting in a pulley at D, and raising the weight E with the band round the head." 236.

By friction, shampooing, and some of the above exercises, the musles of the spine and the ligaments of the vertebræ are rendered more supple, or brought into a very advantageous condition for being stretched. Were we now to raise the patient by a cord and pulley, we should find it much more easily done than before the exercises were used; and if after this the *collar* be applied, much good, in Mr. Shaw's opinion, may be effected. But it will probably be a better plan, after so much fatigue, to let the patient rest—especially if means be employed for the purpose (as may easily be done) of stretching the spinal column, while in the recumbent position. Here Mr. Shaw introduces various proposals and some sketches of apparatus for the purposes in question, but we must refer our readers to the work itself for further particulars.

"In describing the means of preventing or curing slight distortion, I stated the importance of attending to the manner in which a patient lies while asleep; it is still more necessary to attend to it in a case of confirmed curvature of the spine.

"Patients in this condition are desired never to sleep on a feather-bed, but on a firm hair-mattress, and without a pillow. So far, the advice is good, and can be followed; but the order to lie in a particular manner is almost useless; for although a child may be put in a proper position before going to sleep, she will be found in another, in the course of an hour. It being essential that the patient do not lie in a crooked position during the night, some means must be used, by which the pelvis and shoulders may be prevented from approximating. This may be effected by common stiff stays; but the use of the steel supports is preferable, as they may be put on sufficiently tight without compressing the chest. The patient, as may be expected, does not find them comfortable at first, but after they have been used three or four nights seldom complains of them." 253.

Mr. Shaw thinks that, had it not been for the idea so generally prevalent, that lateral distortion depends on ricketts, or on a softening of the bones, the examples daily afforded us of the activity and strength of distorted persons, in the lower ranks of life, would have led to rules of practice very different from those now followed in the treatment of such cases. The dread that deformity of the pelvis would result from the use of instruments is, Mr. Shaw observes, chimerical;—and so is the fear that the bones, in their supposed softened condition, would be twisted by muscular exertion. These erroneous opinions have doubtless prevented many cures, and rendered many a patient miserable for life.

The work concludes with an appendix, containing a short descriptive catalogue of the specimens of distortion and other diseases of the spine, in Mr. Charles Bell's collection.

We need not offer any opinion on Mr. Shaw's work, after the copious extracts, and the general view of it which we have laid before our readers. We imagine that, from this specimen of our author's pathology of the spine, in lateral curvature, the profession will look forward with pleasure to the forthcoming volume on other diseases of the vertebral column.

## Figures and Tables

**Figure f1:**
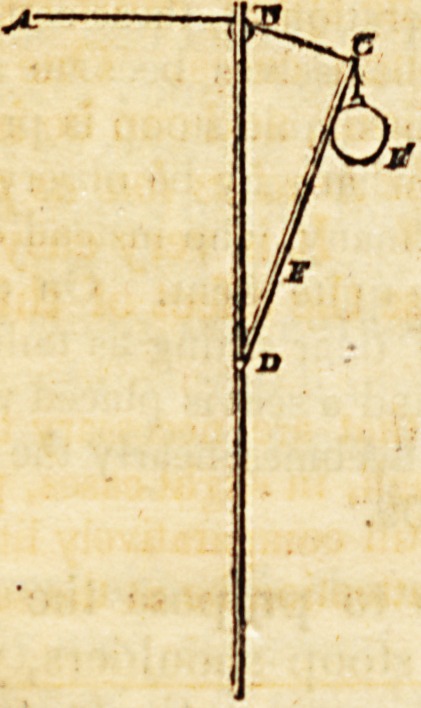


**Figure f2:**
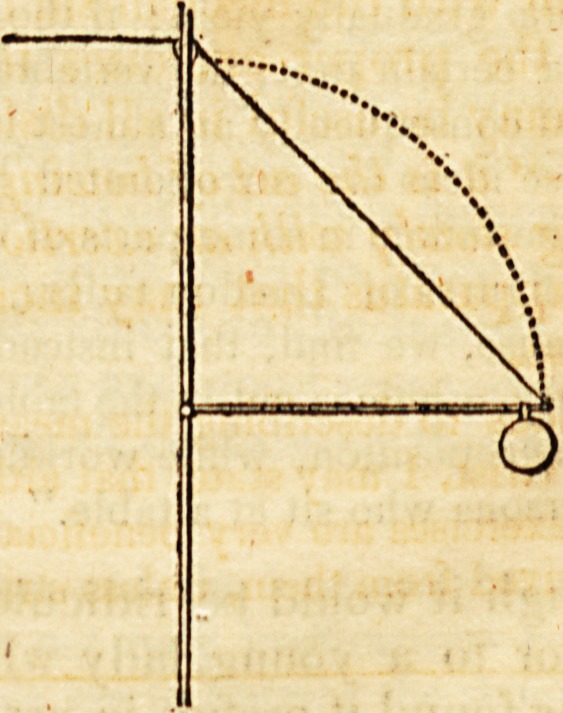


**Fig. 3 Fig. 4 f3:**
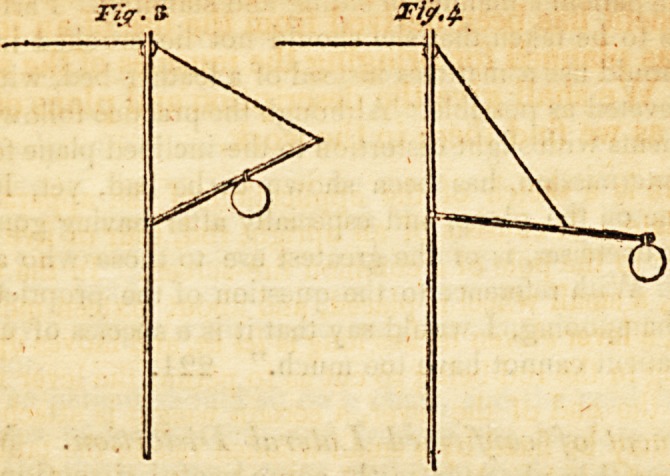


**Figure f4:**
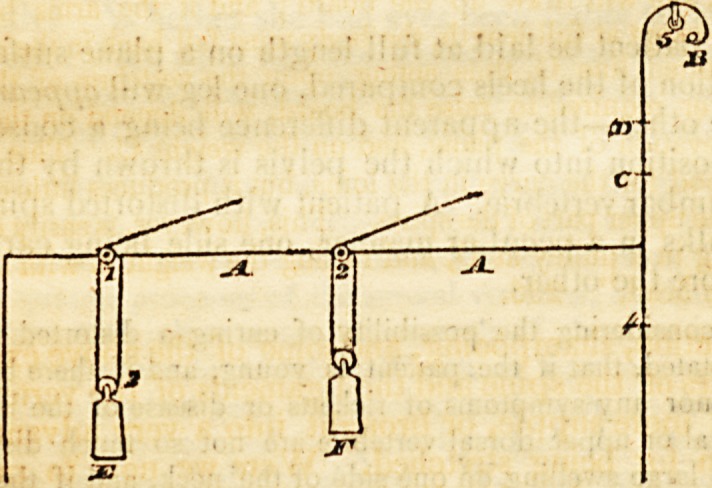


**Figure f5:**